# Identification and functional validation of an enhancer variant in the 9p21.3 locus associated with glaucoma risk and elevated expression of *p16^INK4a^*

**DOI:** 10.1111/acel.13908

**Published:** 2023-06-22

**Authors:** Yizhou Zhu, Cagdas Tazearslan, Michael G. Rosenfeld, Andras Fiser, Yousin Suh

**Affiliations:** ^1^ Department of Obstetrics and Gynecology Columbia University New York New York USA; ^2^ Department of Genetics Albert Einstein College of Medicine Bronx New York USA; ^3^ Department of Medicine School of Medicine University of California La Jolla California USA; ^4^ Howard Hughes Medical Institute University of California La Jolla California USA; ^5^ Department of Systems & Computational Biology Albert Einstein College of Medicine New York New York USA; ^6^ Department of Biochemistry Albert Einstein College of Medicine New York New York USA; ^7^ Department of Genetics and Development Columbia University New York New York USA

**Keywords:** 9p21, cellular senescence, genome‐wide association study, glaucoma, molecular genetics, p16INK4A, YY1

## Abstract

Glaucoma is a leading cause of irreversible blindness, with advanced age being the single most significant risk factor. However, the mechanisms underlying the relationship between aging and glaucoma remain unclear. Genome‐wide association studies (GWAS) have successfully identified genetic variants strongly associated with increased glaucoma risk. Understanding how these variants function in pathogenesis is crucial for translating genetic associations into molecular mechanisms and, ultimately, clinical applications. The chromosome 9p21.3 locus is among the most replicated glaucoma risk loci discovered by GWAS. Nonetheless, the absence of protein‐coding genes in the locus makes interpreting the disease association challenging, leaving the causal variant and molecular mechanism elusive. In this study, we report the identification of a functional glaucoma risk variant, rs6475604. By employing computational and experimental methods, we demonstrated that rs6475604 resides in a repressive regulatory element. Risk allele of rs6475604 disrupts the binding of YY1, a transcription factor known to repress the expression of a neighboring gene in 9p21.3, p16INK4A, which plays a crucial role in cellular senescence and aging. These findings suggest that the glaucoma disease variant contributes to accelerated senescence, providing a molecular link between glaucoma risk and an essential cellular mechanism for human aging.

## Graphical abstract

Although the 9p21.3 non‐coding is a reproducible GWAS locus for glaucoma, the underlying mechanisms by which it contributes to disease risks remain unclear. Here, we report identification of rs6475604 as a functional glaucoma risk variant in the locus. Risk allele of rs6475604 alters binding of YY1, a key transcription factor known for its repression of p16INK4A, and prevents cellular senescence.

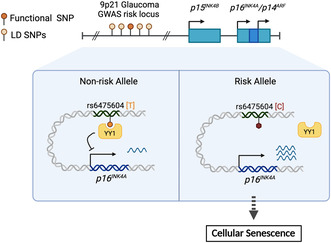

**Abbreviations**
EMSA: gel electrophoresis mobility shift assay
GWAS: genome‐wide association studies
LD: linkage disequilibrium

Glaucoma is a group of optic neurodegenerative diseases that lead to progressive loss of retinal ganglion cells (RGCs) (Weinreb et al., 2014; Weinreb & Khaw, 2004). In the United States and worldwide, glaucoma has emerged as a leading cause of irreversible blindness among older individuals (Parihar, 2016). Numerous studies have identified older age as the most significant and consistent risk factor for glaucoma development (Klein et al., 1992; Leske et al., 1994; Mitchell et al., 1996; Quigley et al., 2001; Wolfs et al., 2000). Despite this correlation, the precise mechanisms through which age‐related pathophysiological changes in RGCs increase disease susceptibility remain largely unknown.

Genome‐wide association studies (GWAS) have consistently identified the 9p21.3 locus as a critical risk factor for glaucoma across various populations (Nakano et al., 2009; Ng et al., 2016; Takamoto et al., 2012; Wiggs et al., 2012). The precise role of this approximately 150 kb non‐coding region in disease risk, however, remains unclear. Epigenetic annotations have uncovered numerous putative regulatory elements within the 9p21.3 locus, pointing to its possible regulatory functions in relation to nearby genes (Harismendy et al., 2011). The INK4 family genes, including CDKN2A (encoding p16^INK4A^ and p14^ARF^) and CDKN2B (encoding p15^INK4B^), which are situated immediately upstream of the locus, play a crucial role in cellular senescence and are considered reliable aging markers in both mice and humans (Jeck et al., 2012; Munoz‐Espin & Serrano, 2014).

Recent studies have demonstrated that increased p16^INK4A^ expression can lead to RGC senescence in cell culture, animal models, and human glaucoma retinas (Skowronska‐Krawczyk et al., 2015). Based on these findings, we propose that the non‐coding glaucoma risk variants within the 9p21.3 locus may have regulatory functions for p16^INK4A^ and could contribute to glaucoma risk by modulating its expression. Unraveling the complex relationship between the 9p21.3 locus, the INK4 family genes, and RGC senescence could provide valuable insights into the underlying mechanisms of age‐related glaucoma and pave the way for the development of novel therapeutic approaches to combat this debilitating disease.

To identify functional variants associated with glaucoma risk in 9p21.3, we obtained the complete list of available glaucoma GWAS from the NHGRI catalogue (Table S1). Six independent studies, including three European and three Japanese populations, have reported six glaucoma‐associated common variants in the 9p21.3 locus. Due to strong linkage disequilibrium (LD) in 9p21.3, multiple variants in the locus were found as significantly associated with glaucoma. Furthermore, the functional variant can be one of the reported GWAS index variants or one in high LD with them. To list the possible candidate functional variants, we examined the LD patterns of the locus using genotyping data from the 1000 Genomes Project. This resulted in an additional 40 candidate variants that were tightly linked (*r* ^2^ > 0.8) to the glaucoma risk variants (Figure 1a). Besides rs1063192, a 3' UTR variant for CDKN2B, all other variants resided in the intronic region of CDKN2B‐AS1, a long non‐coding RNA in the 9p21.3 locus.

**FIGURE 1:**
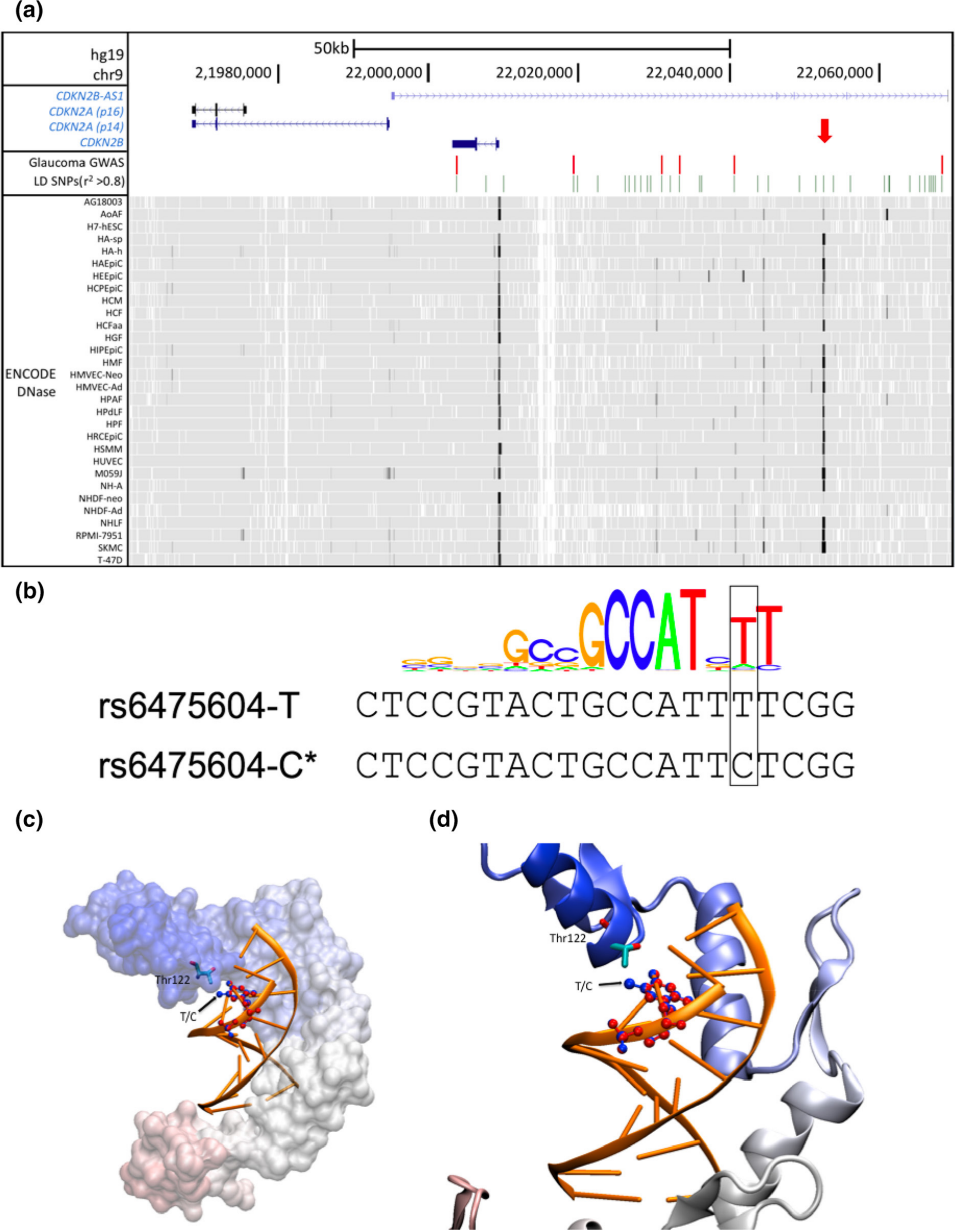
(a) Overview of the 9p21.3 glaucoma risk locus. Locations of glaucoma GWAS risk variants and linked (*r* ^2^ > 0.8) variants (LD SNPs) are indicated in red and green bars, respectively. The bottom DNase hypersensitivity panel from the ENCODE Project suggests putative enhancers in this locus. Red arrow indicates the conserved enhancer that harbors the functional risk variant, rs6475604. (b) Overlay between the consensus motif of human YY1 and the predicted YY1 motif at rs6475604. The glaucoma risk allele (C) disrupts a conserved T nucleotide. (c) Superimposed complex structures of YY1 with two alternative DNA fragments bound to it. The protein model is shown in surface model, colored from red to blue from the N to C terminus. For clarity, the surface model is transparent. The superposed DNA fragments are shown in ribbon models (orange). The only different position T/C is shown in CPK models; blue corresponds to T and red to C nucleic acid base (black arrow). The coordinating Thr122 residue in the YY1 protein is shown in stick model with element based coloring. (d) A close up of panel C, showing the coordinating Thr122 from YY1 in stick model and the nucleic acid base residues in blue (T) and in red (C). Figures were prepared with VMD program.

To narrow down the list of candidate causal variants, we examined epigenomic data from the ENCODE and Roadmap Projects to screen for variants that resided in putative regulatory elements. We found that the 9p21.3 locus was enriched in enhancers, the principal regulatory components of the genome that regulate transcription over a long distance (Zhu et al., 2017). By interrogating the DNase hypersensitive sites for the entire glaucoma LD block (the minimum chromosomal segment containing all LD variants), we found several cell type‐specific weak enhancers, as well as one strong and well‐conserved enhancer that was present in most of the cell lines (Figure 1a). To further consolidate our in silico regulatory function prediction, we annotated the candidate glaucoma risk variants using RegulomeDB, a database that annotates common variants with known and predicted regulatory elements (Boyle et al., 2012). RegulomeDB ranks variants from 1 (most likely) through 6 (least likely) to affect regulatory function. Among all, rs6475604 was the highest‐ranked variant with a score of 2a (Table S2a), which we found to be uniquely located in the identified conserved enhancer (Figure 1a).

The variant rs6475604 is a C/T polymorphism with the C allele associated with the risk of glaucoma. RegulomeDB predicts that rs6475604 is in the canonical and actual binding site of a transcription factor, YY1, and the risk rs6475604‐C allele would interrupt the consensus binding site of YY1 (Figure 1b). We further performed computational modeling of the YY1 protein with both the risk and non‐risk allele. We explored all possible 9‐mer DNA fragments in complex with the YY1 transcription factor using the TF2DNA modeling tool, which uses a structure‐based affinity calculation (Pujato et al., 2014). When ordered by relative binding affinities, the two motifs containing the risk and non‐risk allele (CGCCATTCT and CGCCATTTT) appeared as the 66th and 250th strongest binding DNA fragment among the 262,144 possible combinations (4^9^), indicating that these two fragments were among the most likely binding site candidates (among the top 0.1% of all). Furthermore, the segments CGCCATTTT and CGCCATTCT had relative binding affinities of 487.21 and 465.71, respectively (the higher values indicate stronger binders), demonstrating that the glaucoma risk fragment had reduced binding of YY1 as compared to the non‐risk allele. The two segments differ only by the C/T change in position 8, which is a single methyl group difference in their pyrimidine rings. In the structural model, this extra methyl group in T, the non‐risk allele, appears to interact with the methyl group of Thr122 in the YY1 protein (Figure 1c,d), providing an extra van der Waals interaction and a better overall binding pattern.

Interestingly, YY1 is a general transcription factor known to function as a repressor of p16^INK4A^ gene expression (Wang et al., 2008) and to interact with critical negative regulators of p16^INK4A^, such as polycomb complexes (Wilkinson et al., 2006). If the computational prediction is accurate, the glaucoma risk allele (rs6475604‐C) would increase p16^INK4A^ expression by causing reduced binding of its repressor, YY1. To experimentally test the predicted disruption of YY1 binding by rs6475604‐C compared to the non‐risk rs6475604‐T allele, we performed a competitive gel mobility shift assay (EMSA) using the long YY1 consensus motif (GCCGCCATTTTG) as a biotin‐labeled probe (Kim & Kim, 2009) and the predicted YY1 motif at rs6475604 as unlabeled oligonucleotide competitors for binding of recombinant YY1 (Figure S1). We found that the non‐risk allele (T)‐containing oligonucleotide was a much more effective competitor compared to the risk allele (C), leading to a significant reduction in the intensity of the YY1‐probe complex, especially when applied at 5× concentration (Figure 2a). Notably, the YY1 consensus motif itself (unlabeled probe) showed complete competition under this condition. Reciprocally, we performed the same assay using the predicted YY1 motif containing either the non‐risk or risk allele as labeled probes. The result was consistent, showing that the non‐risk allele competed better for binding of YY1 regardless of the probes used (Figure 2a).

**FIGURE 2:**
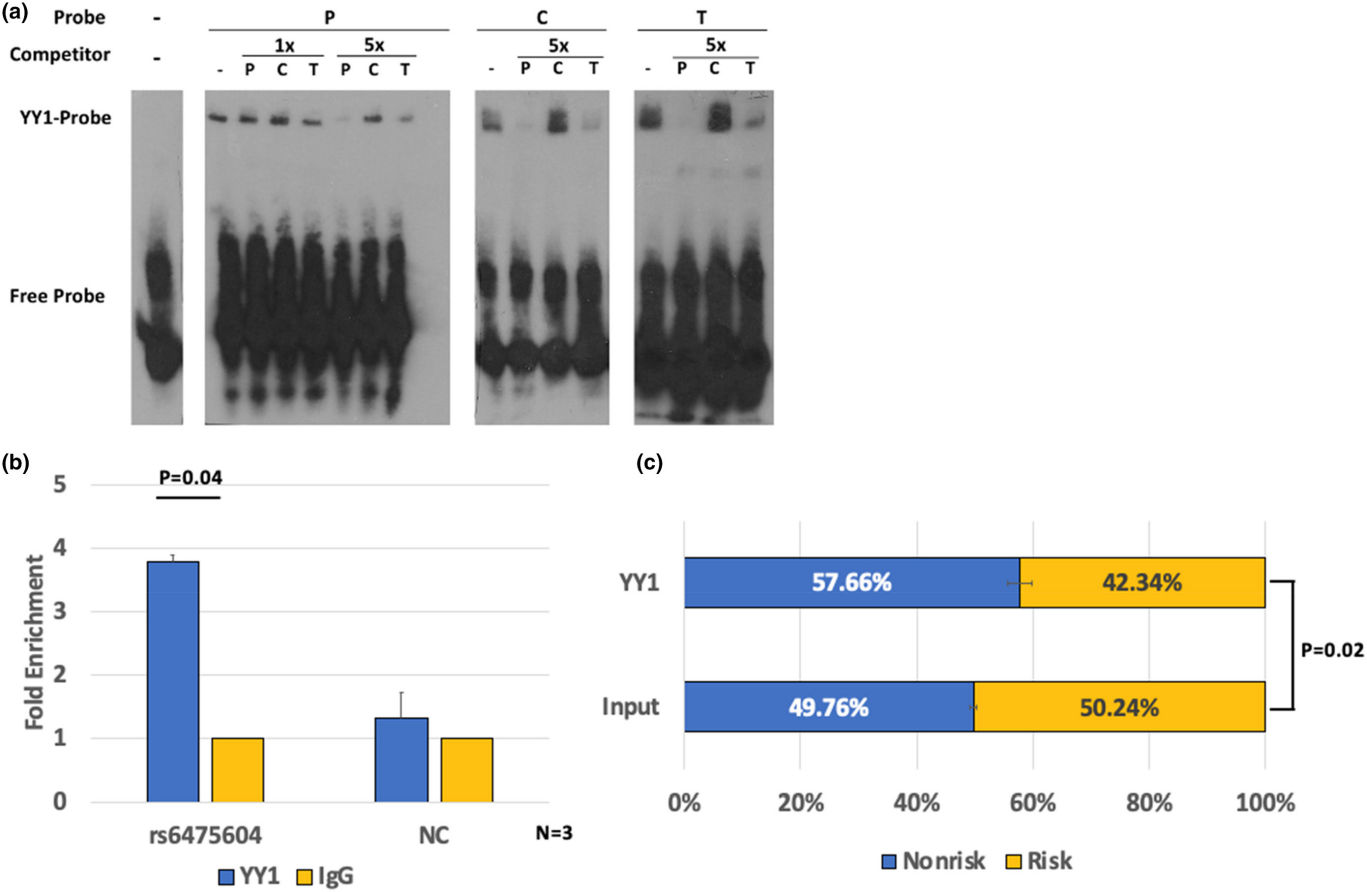
(a) Electrophoretic mobility shift affinity (EMSA) assays of YY1 with rs6475604. P: YY1 consensus motif; C: rs6475604‐C allele; T: rs6475604‐T allele. Binding was performed under no competition (−) or with unlabeled competitors in 1x or 5x concentrations. (b) ChIP‐qPCR and (c) allele‐specific digital PCR of YY1 binding on rs6475604 in neural stem cells differentiated from human embryonic stem cell (H13) (*N* = 3). Negative control (NC) was a nearby locus with no marks of enhancer YY1 binding from ENCODE data. Error bars indicate SEM. Statistics was performed using two‐sided *t* test.

To validate the EMSA result in vivo, we performed ChIP‐qPCR in neural stem cells differentiated from H13 embryonic stem cells. We found that endogenous YY1 significantly bound to the variant locus (3.8‐fold enrichment, Figure 2b). To determine the impact of rs6475604 on YY1 binding, we utilized the heterozygote genotype of the variant in H13 and performed allele‐specific digital PCR, which indicated a significant allelic imbalance of the non‐risk allele over the risk allele (Figure 2c). These observations demonstrated that the glaucoma risk allele (rs6475604‐C) significantly diminished YY1 binding compared to the non‐risk allele (rs6475604‐T), consistent with the in vitro results.

To investigate the functional outcome of the YY1 binding alteration caused by rs6475604, we performed a luciferase reporter assay for the enhancer harboring the variant in four cell lines (Figure S2a). We found that the putative enhancer significantly down‐regulated the reporter in HeLa cells, while showing insignificant trends of down‐regulation in IMR90, MCF7, and HEK293. A comparison of the reporter harboring different alleles of rs6475604 showed that the glaucoma non‐risk allele was consistently associated with stronger reporter repression in all cell lines, although the difference was subtle and insignificant. Taken together, the results suggested that while the rs6475604‐harboring enhancer is likely a repressive regulatory element, its function is cell type and context‐dependent.

Lastly, utilizing the linked glaucoma variant (rs1063192) in the 3'UTR of CDKN2B, we performed allele‐specific qPCR to test whether the alteration of YY1 binding by rs6475604 would affect p15^INK4B^ expression. In H13‐derived NSCs and HEK293, both of which harbored heterozygote 9p21.3 glaucoma genotypes, we did not identify an imbalance between risk and non‐risk alleles (Figure S2b), suggesting that the gene was likely not a target.

In this study, we uncovered a functional variant, rs6475604, located in an enhancer region of the 9p21.3 locus by analyzing and integrating data from GWAS, epigenomics, computational modeling, and experimental validation. Our findings revealed that this enhancer variant led to decreased binding of the YY1 transcription factor, a known repressor of p16INK4A gene expression. This observation aligns with previous studies that demonstrated the recruitment of YY1 to the INK4A‐ARF gene body, along with other polycomb group proteins such as BMI‐1 and EZH2, which negatively regulate p16^INK4A^ expression (Bracken et al., 2007; Itahana et al., 2003; Wang et al., 2008).

The 9p21.3 non‐coding region has been linked to various age‐related disorders apart from glaucoma, including cancers, coronary artery diseases (CAD), and type 2 diabetes (T2D). Fine mapping of risk variants has suggested that the causal mechanisms underlying each disease are likely distinct (Jeck et al., 2012). For CAD, several studies have pointed to p15^INK4B^ or its antisense long non‐coding RNA CDKN2B‐AS1 as the causal effector (Harismendy et al., 2011; Holdt et al., 2013; Zhuang et al., 2012). In contrast, our research indicates that the expression of p15^INK4B^ remains unaltered by the genotype of the glaucoma risk variant, thus presenting p16^INK4A^ as an alternative effector gene.

High expression levels of p16^INK4A^ are crucial for inducing cellular senescence (Coppe et al., 2011; Rayess et al., 2012). As organisms age, senescent cells accumulate and contribute to numerous age‐related diseases such as osteoarthritis, cataracts, tumorigenesis, cardiac hypertrophy, renal dysfunction, lipodystrophy, and sarcopenia in mice (Childs et al., 2017). Previous studies have reported increased p16^INK4A^ expression in glaucomatous eyes in mice and humans, with elevated p16^INK4A^ levels being causally connected to RGC senescence in mouse models (Skowronska‐Krawczyk et al., 2015). Furthermore, the glaucoma risk genotype in SIX6, an activator of p16^INK4A^ expression, has been associated with higher p16^INK4A^ expression and senescence in human RGCs (Skowronska‐Krawczyk et al., 2015). In light of these findings, our data suggest that genetic variants that increase p16^INK4A^ expression may contribute to glaucoma risk by enhancing the propensity for cellular senescence, a fundamental aging mechanism, in humans. This understanding could potentially inform future therapeutic strategies targeting cellular senescence in glaucoma diseases.

## AUTHOR CONTRIBUTIONS

Conceptualization: Yizhou Zhu, Yousin Suh; Methodology: Yizhou Zhu, Cagdas Tazearslan, Yousin Suh; Analysis: Yizhou Zhu, Andras Fiser; Drafting: Yizhou Zhu, Yousin Suh; Editing: all authors.

## FUNDING INFORMATION

This work was supported by NIH grants AG017242, AG076040, AG061521, and AG056278 (Y.S), a grant GCRLE‐1320 (Y.S.) from the Global Consortium for Reproductive Longevity and Equality at the Buck Institute, made possible by the Bia‐Echo Foundation, and a grant from The Simons Foundation (Y.S.).

## CONFLICT OF INTEREST STATEMENT

The authors declare no conflict of interest.

## Supporting information


Data S1.

## Data Availability

Data are available on request from the authors.
