# Supplementary material for: Identification and functional validation of an enhancer variant in the 9p21.3 locus associated with glaucoma risk and elevated expression of p16INK4a
Source: Aging Cell. 2023 Jun 22;22(9):e13908. doi: 10.1111/acel.13908 (PMC10497822; doi:10.1111/acel.13908)
Supplement: Supplementary file 1 — Data S1. [file ACEL-22-e13908-s001.zip › acel13908-sup-0001-Data.pdf]

Experimental Procedures

SNP selection

SNP Selection The list of glaucoma-associated GWAS variants was obtained from the GWAS Catalogue (https://www.ebi.ac.uk/gwas/, as of November 2017), a comprehensive database for GWAS that currently includes 2,854 publications and 33,674 unique SNP-trait associations. The default filter for p-value ($p<5x10^{-8}$) was used to search for significant associations, and no additional filter on effect size was applied. Variants were considered in the 9p21.3 locus if they were located within the coordinates, chr9:21950000-22180000 (hg38).

Linkage Disequilibrium (LD) analysis

We used SNAP (SNP Annotation and Proxy Search) to examine the reported GWAS variants to obtain a full list of SNPs in LD with the 9p21 glaucoma variants (Johnson et al. 2008). Variants with LD r^2 >= 0.8 were included for downstream analysis of in silico function prediction. The variants found in the 1000 Genomes and HapMap Projects were combined to obtain the final list, after removing redundancies. For determining the risk allele for LD variants, we constructed haplotypes using Haploview (Barrett et al. 2005) and the individual genotype information of the 1000 Genomes Project.

RegulomeDB

RegulomeDB is a bioinformatics tool for predicting the regulatory function of ~60 million annotated variants (Boyle et al. 2012). The database is built upon 962 epigenomic datasets from the ENCODE consortium, NCBI Sequence Read Archive, and other sources, including a large collection of eQTL studies. The database ranks variants from 1 (highest) to 6 (lowest) for their likelihood of exerting regulatory functions. Rank 1 and 2 variants are considered likely to be functional based on strong evidence from multiple datasets. The tool is freely accessible at http://www.regulomedb.org. We applied RegulomeDB to all 9p21 glaucoma variants and GWAS variants to examine potential regulatory functions.

Electrophoresis mobility shift affinity (EMSA) assay

The following sequences were used for the gel mobility shift assay:
YY1-consensus: CGCTCCGT**GCCGCCATTTTG**GGCGGCTGGT
rs6475604-C[Risk]: CGCTCCGT**ACTGCCATTCTC**GGCGGCTGGT
rs6475604-T[Non-Risk]: CGCTCCGT**ACTGCCATTTTC**GGCGGCTGGT

The 3'-biotinylated probes and unlabeled competitors were synthesized by IDT and annealed with the corresponding reverse complements. Forty nanograms of probes were mixed with 200 ng recombinant YY1 (31332, Active Motif) in a buffer containing 10 mM Tris-HCl (pH 7.4), 150 mM KCl, 0.1 mM DTT, and 0.1 mM EDTA, and incubated for 20 min at room temperature. For competition binding assays, unlabeled competitors with 1X and 5X concentrations were pre-incubated with YY1 protein before adding labeled probes. The incubated protein-DNA complex was loaded on a 6% polyacrylamide gel and run at 100 V for 50 min in 1x TBE buffer. The gel was then transferred to a nitrocellulose

membrane and probed with streptavidin-HRP (Abcam ab7403) and imaged on exposure film (Thermo 34090) using ECL substrate(Thermo 32106).

In silico calculation of relative binding affinities
Relative binding affinities were obtained using the TF2DNA algorithm (Pujato et al. 2014). Briefly, TF2DNA construct a three dimensional computational homology model of the complex of the protein transcription factor and cognate DNA fragment and assesses the relative binding affinity using a knowledge-based statistical pair potential, after optimization of the complex in a molecular mechanics force field. In case of YY1 transcription factor 9 residue long, double stranded DNA fragments were explored in all possible combinatorial variation, resulting in (49)=262,144 different complexes and corresponding energies.

Cell Culture
Human embryonic stem cells H13 (WiCell WA13) were maintained in a feeder-free E8 system (ThermoFisher A1517001). To differentiate into neural stem cells, ES cells were sub-cultured at 15% confluency, and cultured in PSC Neural Induction Medium (Thermo A1647801) for 6 days as the manufacturer indicated. After reaching confluency, cells were maintained in neural expansion medium (49% Neurobasal medium, 49% Advanced DMEM-F12, 2% neural induction supplement) for two passages before collection.

Allele-Specific digital PCR
RNA was isolated using the PureLink RNA Mini Kit (ThermoFisher) according to the manufacturer's instructions. DNase treatment was performed using the DNA-free DNase Treatment & Removal Kit (ThermoFisher). To synthesize cDNA, reverse transcription was performed using SuperScript IV Reverse Transcriptase (ThermoFisher) with oligo-dT primers, following the manufacturer's instructions.

To perform allele-specific expression analyses of CDKN2B, 50 ng of cDNA was loaded onto the Quantstudio 3D digital PCR system (version 2, ThermoFisher) and amplified using Taqman SNP arrays (rs1063192, ThermoFisher). Allele-specific signal quantification was performed using the online cloud application provided by the manufacturer.

Chromatin immunoprecipitation
For crosslinking, 10 million NSCs were crosslinked using 1% formaldehyde (Thermo 28908) in PBS for 10 min. The reaction was quenched by incubating in 125 mM glycine for 5 min, and cells were washed with PBS twice and harvested using a silicone scraper. To create nuclear lysate, cells were resuspended sequentially in 10 mL Buffer 1 (50 mM HEPES-KOH pH 7.5, 10 mM NaCl, 1 mM EDTA, 10% glycerol, 0.5% IGEPAL CA-630, 0.25% Triton X-100), 10 mL Buffer 2 (10 mM Tris-HCl, pH 8.0, 200 mM NaCl, 1 mM EDTA, 0.5 mM EGTA), and 900 µl Buffer 3 (10 mM Tris-HCl, pH 8.0, 100 mM NaCl, 1 mM EDTA, 0.5 mM EGTA, 0.1% Na-Deoxycholate, 0.5% N-lauroylsarcosine). In Buffer 3, the lysate was sonicated using a Bioruptor (Diagenode, 12 min at high energy output, 30 sec on, 30 sec off), and mixed with 10 µg YY1 antibody (Santa Cruz sc-7341) or IgG (Thermo 10400C) in 100 µl Protein G beads (Thermo 10003D) for 14 hr

immunoprecipitation at 4 °C. The beads were cleaned up with 2x washes of low salt buffer (20 mM Tris-HCl, pH 7.5, 150 mM NaCl, 2 mM EDTA, 0.1% SDS, 1% Triton-X100), 2x of high salt buffer (20 mM Tris-HCl, pH 7.5, 500 mM NaCl, 2 mM EDTA, 0.1% SDS, 1% Triton-X100), and 1x LiCl buffer (10 mM Tris-HCl, pH 7.5, 250 mM LiCl, 1 mM EDTA, 1% Na-Deoxycholate, 1% IGEPAL CA-630). The bead-bound complex was eluted with 200 µl elution buffer (50 mM Tris-HCl, pH 8.0, 10 mM EDTA, 1% SDS) for 30 min at 65°C. The eluent was sequentially treated with 0.2 mg/mL RNase A and 0.2 mg/mL Proteinase K, and de-crosslinked at 68°C for 14 hours. The DNA content was recovered using phenol-chloroform separation, and resuspended in 20 µl of 10 mM Tris-HCl, pH 8.0. For quantitative and allele-specific analysis, 7.6 µl of the final product was mixed with 8 µl of 2x QuantStudio 3D Digital PCR Master Mix v2 (Thermo A26358) and 0.4 µl Taqman SNP primer for rs6475604 (C___1754703_10) or rs1333040 (C___8766795_10) for negative control. The mixture was loaded onto the QuantStudio 3D Digital PCR system (Thermo) and analyzed using the provided cloud analysis system, following the manufacturer's instructions.

Luciferase reporter Assay

The enhancer region harboring rs6475604 (chr9: 22050310-22054084, hg38) was amplified from human mixed DNA (Promega G3041) using the following primer pairs: Fw: AAATCGATAAGGATCCAGGCCTTGCAATTGATTACG, Rev: AAGGGCATCGGTCGAC GCACAAGAAATGCTAGCTAAGG, and cloned into the pGL4.23 backbone (Promega E8411) at the BamHI/SalI site using the In-Fusion cloning tool (Clontech). The product was transformed into Stellar competent cells (Clontech) to select clones with successful integration. After confirming the sequence using Sanger sequencing, desired clones were purified using the PureLink HiPure Plasmid Midiprep Kit (ThermoFisher). Five hundred nanograms of vector were co-transfected with 25 ng CMV-Renilla (Promega E2261) into subject cell lines in 24-well plates at 70% confluence using Lipofectamine 3000 reagent. After 24 hours, cells were analyzed using the Dual-Luciferase Reporter Assay System (Promega E1960) according to the manufacturer's protocol. The reporter activity fold change was calculated with dual normalization against the empty pGL vector and Renilla reading. Luminescence was measured on a microplate luminometer (SpectraMax, Molecular Devices).

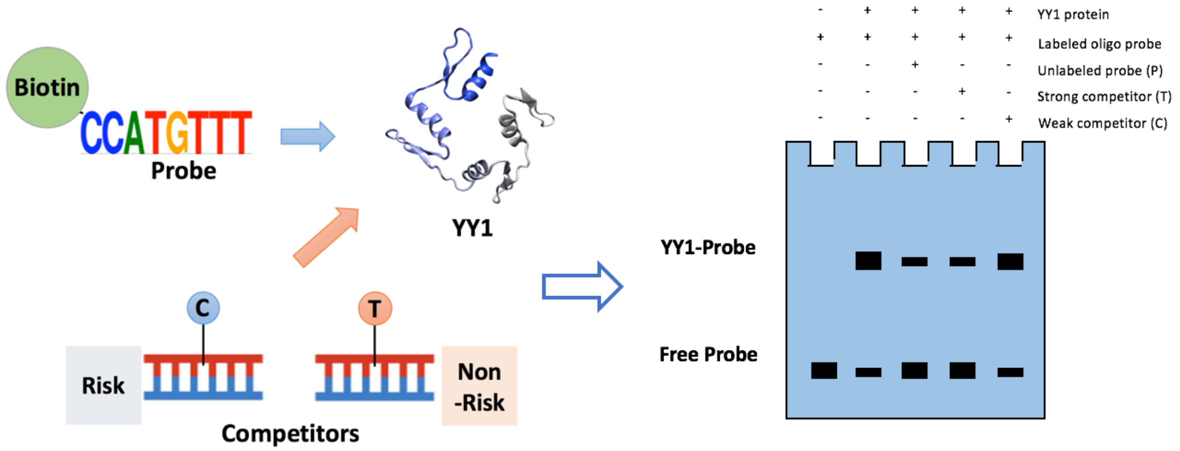

Figure S1. Schematic for the EMSA design and expected outcome from the competitive EMSA assay based on the computational prediction.

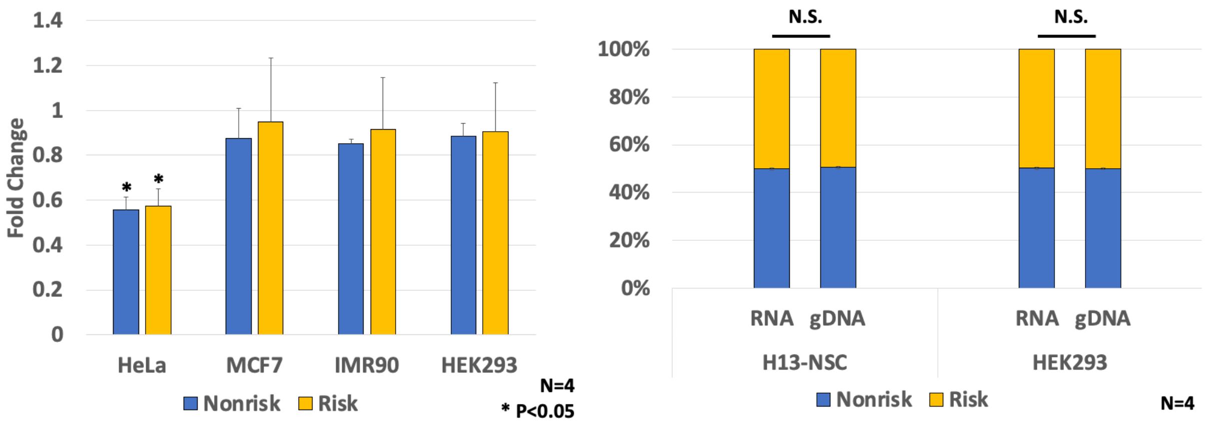

Figure S2. (A) Luciferase activity of rs6475604 enhancer region carrying non-risk or risk allele of the variant. (B) Allele-specific dPCR for CDKN2B expression in HEK293 and H13-derived neural stem cell.

SNP ID	SNP POS	Ethinic Group	Location	Sample Size	p-value	Study
rs1063192-T	chr9:22003368	Japanese	3' UTR	1394 Case, 6599 Controls	5E-11	Osman W
rs523096-A	chr9:22019130	Japanese	Intronic	286 Cases, 557 Controls	5E-11	Takamoto M
rs7865618-A	chr9:22031006	Japanese	Intronic	833 Cases, 686 Controls	9E-11	Nakano M
rs2157719-?	chr9:22033367	European	Intronic	3146 Cases, 3487 Controls	2E-18	Wiggs JL
rs1333037-T	chr9:22040766	European	Intronic	725 Cases, 11145 Controls	1E-12	Cooke Bailey JN
rs4977756-A	chr9:22068653	European	Intronic	1155 Cases, 1922 Contols	7E-30	Burdon KP Gharahkhani P

Table S1. Reported GWAS variants associated with glaucoma in 9p21.3

SNP ID	Chr	Pos	Reglome Score
rs6475604	chr9	22052733	2a
rs3217977	chr9	22007357	3a
**rs523096**	chr9	22019128	3a
**rs1333037**	chr9	22040764	4
rs200059580	chr9	22049479	4
rs2069418	chr9	22009697	4
**rs1063192**	chr9	22003366	5
rs10811648	chr9	22067541	5
rs10811649	chr9	22067553	5
rs10811651	chr9	22067829	5
rs10965224	chr9	22067275	5
rs1333039	chr9	22065656	5
rs1412829	chr9	22043925	5
rs142048183	chr9	22028212	5
**rs2157719**	chr9	22033365	5
rs2383205	chr9	22060934	5
**rs4977756**	chr9	22068651	5
rs581876	chr9	22022375	5
rs61271866	chr9	21997014	5
rs634537	chr9	22032151	5
rs679038	chr9	22029079	5
rs1008878	chr9	22036111	6
rs10965223	chr9	22067003	6
rs4451405	chr9	22071749	6
rs4977755	chr9	22066362	6
rs564398	chr9	22029546	6
rs599452	chr9	22027401	6
rs615552	chr9	22026076	6
rs7866783	chr9	22056358	6
rs1360589	chr9	22045316	7
rs1537378	chr9	22061613	7
rs1556515	chr9	22036366	7
rs2184061	chr9	22061561	7
rs518394	chr9	22019672	7
rs543830	chr9	22026638	7
rs613312	chr9	22026593	7
rs7030641	chr9	22054039	7
**rs7865618**	chr9	22031004	7
rs8181050	chr9	22064390	7
rs944801	chr9	22051669	7

Table S2. RegulomeDB score of 9p21 glaucoma risk variants (r^2 > 0.8). Score 7 is given to variants no available annotation data.
